# Comparative transcriptomics reveals different strategies of *Trichoderma* mycoparasitism

**DOI:** 10.1186/1471-2164-14-121

**Published:** 2013-02-22

**Authors:** Lea Atanasova, Stephane Le Crom, Sabine Gruber, Fanny Coulpier, Verena Seidl-Seiboth, Christian P Kubicek, Irina S Druzhinina

**Affiliations:** 1 Research Area Biotechnology and Microbiology, Institute of Chemical Engineering, Vienna University of Technology, Gumpendorferstrasse 1a, A-1060, Vienna, Austria; 2 École normale supérieure, Institut de Biologie de l’ENS, IBENS, F-75005, Paris, France; 3 Inserm, U1024, F-75005, Paris, France; 4 CNRS, UMR 8197, F-75005, Paris, France; 5 UPMC Univ Paris 06, UMR7622, Laboratoire de Biologie du Développement, 9 quai St. Bernard, F-75005, Paris, France; 6 CNRS, UMR7622, Laboratoire de Biologie du Développement, 9 quai St. Bernard, F-75005, Paris, France; 7 Austrian Center of Industrial Biotechnology (ACIB), GmBH c/o Institute of Chemical Engineering, Vienna University of Technology, Gumpendorferstrasse 1a, A-1060, Vienna, Austria

**Keywords:** *Hypocrea*, *T. atroviride*, *T. virens*, *T. reesei*, Mycoparasitism, Gene expression, Biocontrol, Transcriptomics

## Abstract

**Background:**

*Trichoderma* is a genus of mycotrophic filamentous fungi (teleomorph *Hypocrea*) which possess a bright variety of biotrophic and saprotrophic lifestyles. The ability to parasitize and/or kill other fungi (mycoparasitism) is used in plant protection against soil-borne fungal diseases (biological control, or biocontrol). To investigate mechanisms of mycoparasitism, we compared the transcriptional responses of cosmopolitan opportunistic species and powerful biocontrol agents *Trichoderma atroviride* and *T. virens* with tropical ecologically restricted species *T. reesei* during confrontations with a plant pathogenic fungus *Rhizoctonia solani*.

**Results:**

The three *Trichoderma* spp. exhibited a strikingly different transcriptomic response already before physical contact with alien hyphae. *T. atroviride* expressed an array of genes involved in production of secondary metabolites, GH16 ß-glucanases, various proteases and small secreted cysteine rich proteins. *T. virens*, on the other hand, expressed mainly the genes for biosynthesis of gliotoxin, respective precursors and also glutathione, which is necessary for gliotoxin biosynthesis. In contrast, *T. reesei* increased the expression of genes encoding cellulases and hemicellulases, and of the genes involved in solute transport. The majority of differentially regulated genes were orthologues present in all three species or both in *T. atroviride* and *T. virens*, indicating that the regulation of expression of these genes is different in the three *Trichoderma* spp. The genes expressed in all three fungi exhibited a nonrandom genomic distribution, indicating a possibility for their regulation via chromatin modification.

**Conclusion:**

This genome-wide expression study demonstrates that the initial *Trichoderma* mycotrophy has differentiated into several alternative ecological strategies ranging from parasitism to predation and saprotrophy. It provides first insights into the mechanisms of interactions between *Trichoderma* and other fungi that may be exploited for further development of biofungicides.

## Background

Mycoparasitism describes the type of biotrophic interactions in which organisms benefit at the expense of the fungi [1]. In a broad sense this property is most proliferated within the fungal family *Hypocreaceae,* and the ability to antagonize, parasitize or even kill other fungi (necrotrophic hyperparasitism, mycotrophy) is particularly common in the genus *Trichoderma* (teleomorph *Hypocrea*, Hypocreales, Dikarya) [1]. The biochemistry and genetics of mycoparasitism has been most thoroughly investigated in only a few species of *Trichoderma* such as *Trichoderma harzianum* sensu lato*, T. atroviride* (teleomorph *Hypocrea atroviridis*)*, T. virens* (teleomorph *H. virens*)*, T. asperellum* and *T. asperelloides*[1-3] because of their application as agents of biological control of pests (biocontrol) in agriculture*.* Consequently, several enzymes and other effectors involved in the recognition of a fungus and in the mycoparasitic responses itself have been identified [4-7]. Expressed-sequence-tag (EST) libraries obtained of different *Trichoderma* strains cultivated under various conditions have contributed significantly to the large-scale identification of genes required for the interaction [8-12]. DNA microarrays have been used to study the interaction of *Trichoderma* with plants [13]. So far, however, only two studies used high throughput transcriptomic tools to investigate mechanisms of *Trichoderma* mycoparasitism [5,11].

A comparative analysis of the genome inventory of *T. atroviride, T. virens* and *T. reesei* revealed that mycoparasitism is the innate property of the genus *Trichoderma*[1,4]. It has been suggested that the evolutionary more recent species *T. reesei* adapted to a certain ecological niche and thereby partially lost the strength of its mycoparasitic potential [1,4]. A comparison of genome wide gene expression in vigorous and moderate mycoparasitic *Trichoderma* species may therefore lead to the identification of mechanisms involved in the interaction.

In this study we have applied oligonucleotide tiling microarrays to obtain transcriptional profiles of *T. atroviride* IMI 206040, *T. virens* Gv29-8 and *T. reesei* QM 6a (which were used for genome sequencing) interaction with the plant pathogenic fungus *Rhizoctonia solani* (teleomorph *Thanatephorus,* Basidiomycota). Our data highlight the differences in the mechanisms of these interactions between *Trichoderma* species and emphasize the existence of alternative strategies employed by these fungi.

## Results

### Confrontation assays and *Trichoderma* mode of action

**Figure 1:**
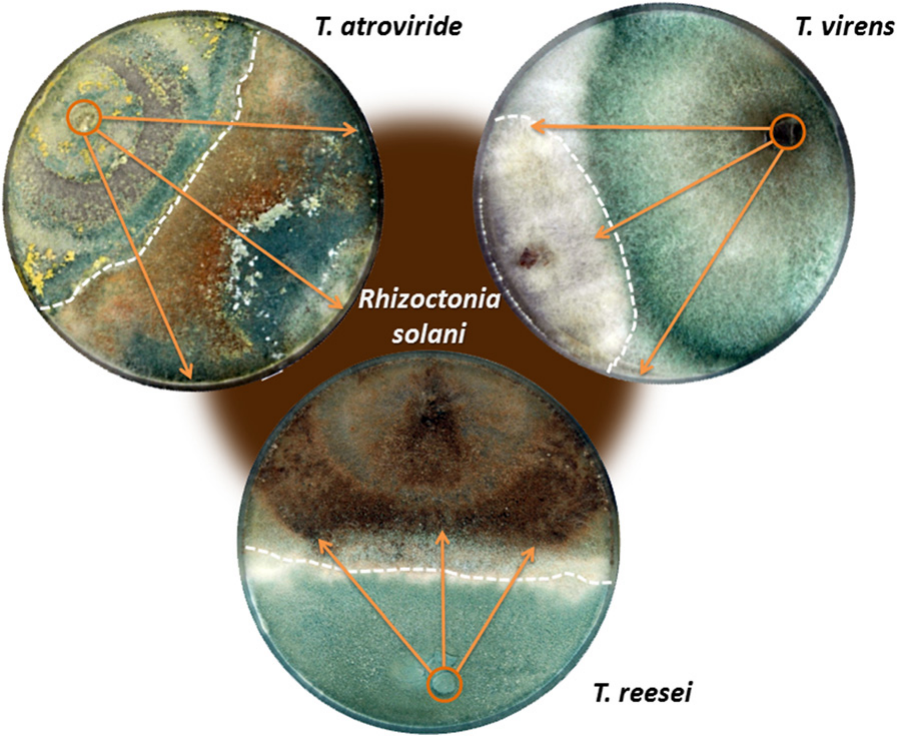
**Mycoparasitism of *Trichoderma *spp. in dual confrontations assays with *Rhizoctonia solani.*** Fungi were incubated for 10 days on PDA at 25°C and 12 hours cyclic illumination. Arrows indicate overgrowth of *R. solani* by *Trichoderma* spp*.*

In order to identify the genes that are involved in *T. atroviride* (*Ta*), *T. virens* (*Tv*) and *T. reesei* (*Tr*) interaction with *R. solani*, we performed dual confrontation assays. *R. solani,* a plant pathogenic fungus was chosen as a model prey fungus because several biocontrol formulations based on *Trichoderma* target various plant diseases caused by this fungus [14] and as it makes severe agricultural damages on a wide range of crops worldwide [15]. The three *Trichoderma* spp. revealed essential differences in their response to confrontation with *R. solani* (Figure 1): *Tr* was at first unable to stop the growth of *R. solani*, and the growth of the latter one was even stimulated for 21% more than when confronted to itself. However, after 10 days at the borderline between the two cultures there was a clear barrage zone that was later consequently overgrown by *Tr* (Figure 1). *Tv* inhibited the growth of *R. solani* by 9%, but it was able to fully overgrow *R. solani* colony and finally killed it. In contrast*,* at 10th day *Ta* did not stop the growth of *R. solani*, but it straightforwardly overgrew it almost completely (86%, Figure 1). Thus, in terms of this experiment, *Tv* and *Ta* expressed interactions best described as predation (immediate kill and consumption) and parasitism respectively. *Tr* showed neutral reaction with signs of weak mycoparasitism.

### Transcriptional response of *Trichoderma* to the interaction with *R. solani*

**Figure 2:**
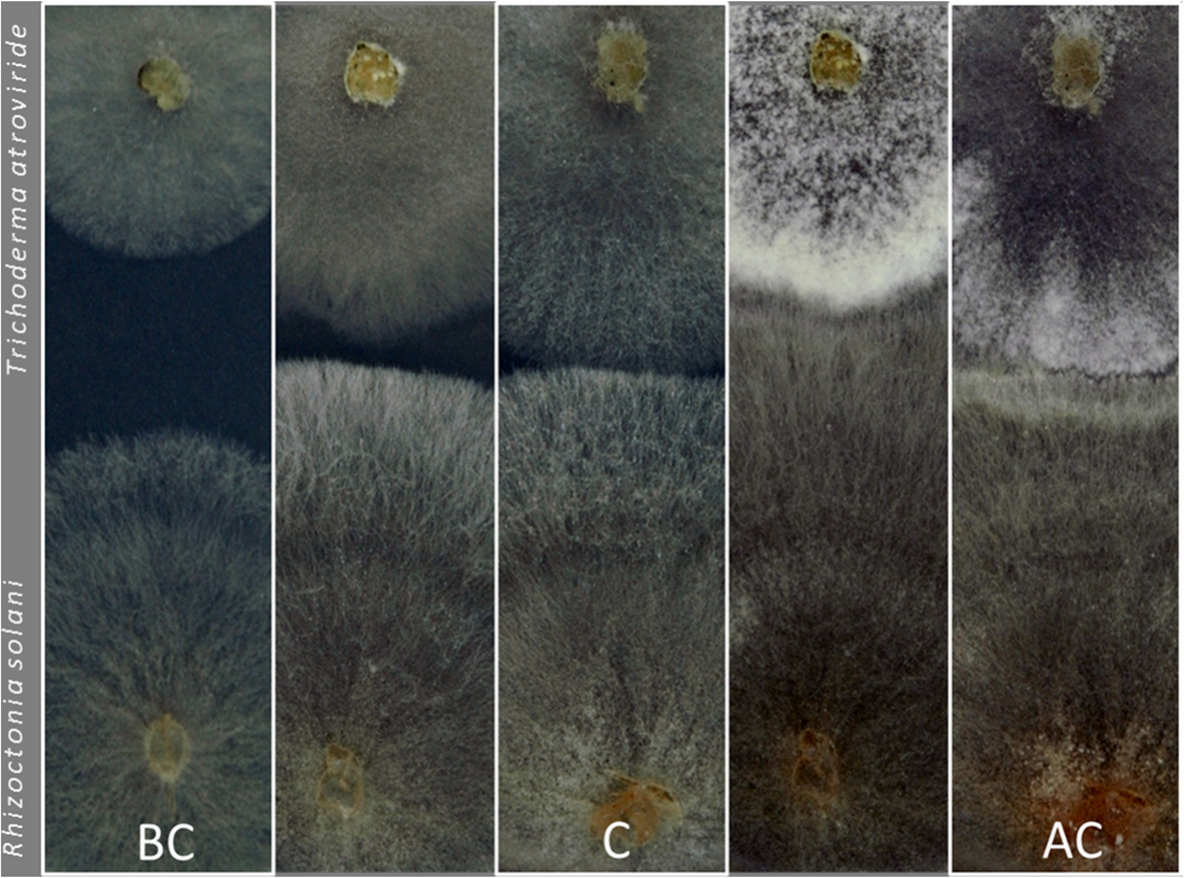
**Development of mycoparasitic reaction of *Trichoderma atroviride *against *Rhizoctonia solani*.** The mycelium was sampled before contact (**BC**), at the contact (**C**) and after the contact (=overgrowth, **AC**) with *R. solani*.

**Figure 3:**
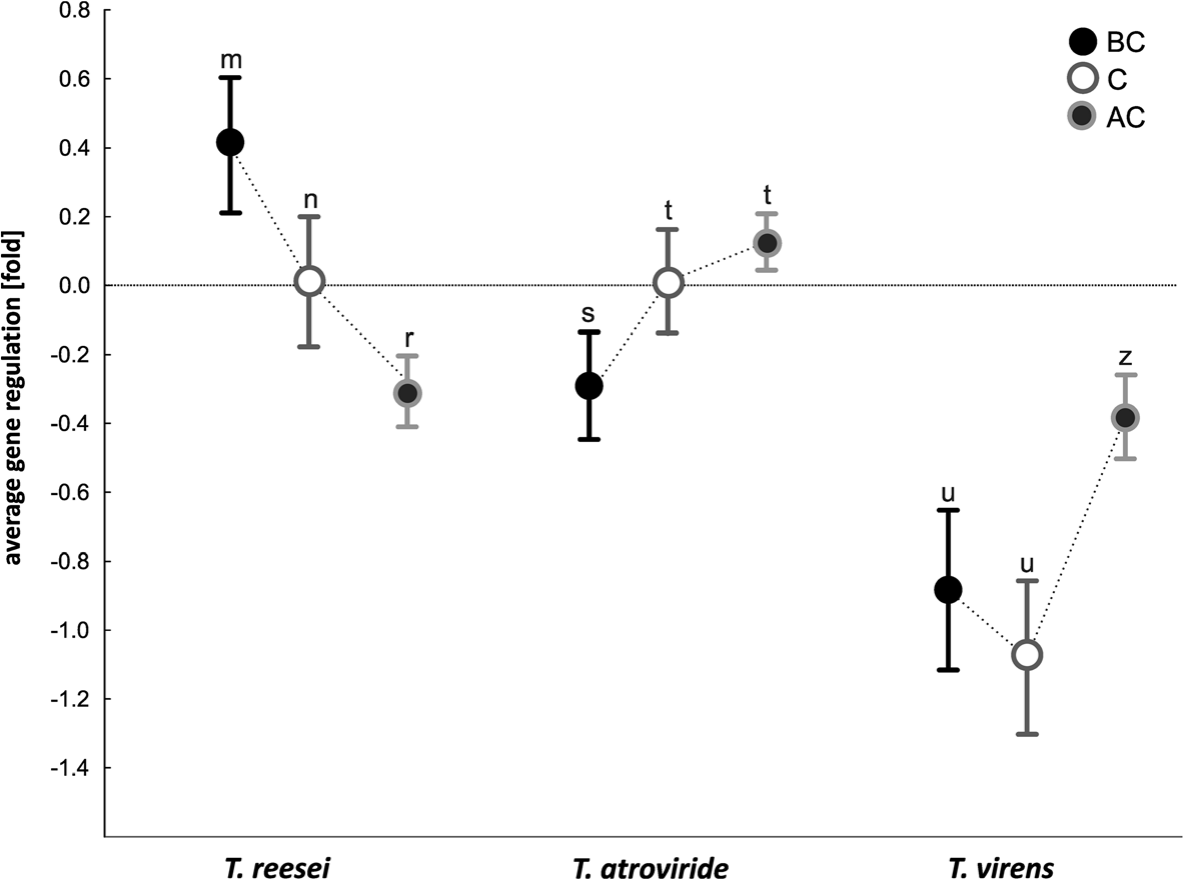
**Averaged regulation of all genes (|log**_**2 **_**(ratio)| > 1.5) involved in mycoparasitsm of *T. reesei*, *T. atroviride *and *T. virens *during the three confrontation stages with *R. solani *(BC, C and AC).** Vertical bars denote 0.95 confidence intervals. The same letters above the bars indicate the statistical significance (ANOVA) between the stages of each species: for m–n–r p < 0.005; s–t p < 0.02; u–z p < 0.001. The plot shows different trends of gene expression kinetics between the species and does not reflect the gene regulation intensity differences among the species.

The tiling microarray data of *Ta, Tv* and *Tr* were analyzed at three stages of the interaction: (1) before contact with alien hyphae (BC), (2) during the initial contact with the hyphae (C), and (3) when the interaction has been established (after contact; AC) (Figure 2). After data normalization we applied the linear modeling approach and the Bayes statistics implemented in the LIMMA package (http://bioinf.wehi.edu.au/limma/, [16]) to the biological replicates as described in Materials and Methods. In total more than 60% of the genes present in the three genomes were found to be transcribed. Among them 651, 303 and 424 genes were found to be differentially regulated (larger/smaller than log_2_ (ratio) = 1.5) in *Ta*, *Tv* and *Tr*, respectively (Table 1; Additional file 1: Tables S1-S3) and thus involved in the interaction. All of up- and down-regulated genes were annotated and categorized as described in the Materials and Methods section. In addition, Functional Catalogue (FunCat, http://mips.helmholtz-muenchen.de/proj/funcatDB/) protein families were assigned to those genes for which a function could be predicted (Table 2). 66, 70 and 62% of the significantly expressed genes of *Ta*, *Tv* and *Tr*, respectively, encoded genes with significant similarity to other fungal proteins (blastp E < e^-100^; Table 1). The remaining genes were annotated as unknown (when orthologues were found in other fungal genera but no function was assigned) or orphan (unique; present only in *Trichoderma* spp.) proteins (see Materials and Methods for definition). The latter comprised only 0.5-0.7% of all transcripts for the three *Trichoderma* species (Table 1). Statistical analysis revealed that the transcriptomic profiles of three investigated species were significantly different (ANOVA, p < 0.001). The majority of involved genes in *Tr* and *Tv* (63 and 84% respectively) were down-regulated*,* whereas the up- and down-regulated genes in *Ta* occurred in approximately equal proportions (52% up- and 48% down-regulated). However, in all species the pattern of up- and down-regulation depended on the confrontation stages (Figure 3).

**Table 1:** **Numbers of transcripts that are either up- or down-regulated during the mycoparasitic response of *T. reesei*, *T. atroviride *and *T. virens***

**Species**	**Section**	**Genes**	**Transcripts**		**Proteins**					
					**With identified function**		**Unknown**		**Orphan**	
			**Total**	Up/down (%*)	**Total**	**Up/down (%)**	**Total**	**Up/down (%)**	**Total**	**Up/down (%)**
*T. reesei*	*Longibrachiatum*	9 143	424	36.6/**63.4**	264	44.7/**55.3**	137	23.4/**76.6**	23	21.7/**78.3**
*T. atroviride*	*Trichoderma*	11 865	651	**53.1**/46.9	432	**52.1**/47.9	173	**58.4**/41.6	46	43.5/**56.5**
*T. virens*	*Pachybasium*	12 428	303	15.5/**84.5**	211	19.4/**80.6**	71	8.5/**91.5**	21	0/**100**

*the percentages of up- and down regulated genes are calculated based on the total number of transcripts found within each group of genes; bold fond highlights larger values.

**Table 2:** Protein groups (FunCat) that exhibited significant up- and/or down-regulation during mycoparasitic interaction in at least one of the three *Trichoderma *spp.*

**FunCat category**	**FunCat number**	**Annotation**	***T. atroviride***				***T. virens***				***T. reesei***			
			**11 865 genes**				**12 428 genes**				**9 143 genes**			
			**Total involved**	**Up down**		**Up/down (%)**	**Total involved**	**Up down**		**Up/down (%)**	**Total involved**	**Up down**		**Up/down (%)**
metabolism	01_05_01_01_09	2-oxoglutarate dependent dioxygenase	*3*	*0*	*3*	0	*1*	*1*	*0*	n.a.	*0*	*0*	*0*	n.a.
	01_04	acid phosphatase	*4*	*4*	*0*	n.a.	*1*	*1*	*0*	n.a.	*1*	*1*	*0*	n.a.
	01_20_05_11	PKS	*5*	*2*	*3*	0.7	*1*	*0*	*1*	0	*4*	*2*	*2*	1
	01_20_35_01_05	flavonol reductase	*4*	*4*	*0*	n.a.	*1*	*0*	*1*	0	*0*	*0*	*0*	
	01_20_35_01_05	isoflavon reductase	*2*	*2*	*0*	n.a.	*0*	*0*	*0*	0	*1*	*0*	*1*	0
	01_20_36	NRPS	*2*	*0*	*2*	0	*4*	*0*	*4*	0	*2*	*0*	*2*	0
	01_25_01	GH glycosyl hydrolase	*51*	*10*	*41*	0.2	*5*	*4*	*1*	4	*36*	*34*	*2*	17
	01_25_03	protease	*15*	*13*	*2*	6.5	*3*	*1*	*2*	0.5	*5*	*2*	*3*	0.7
	01_25_07	lipase/esterase	*6*	*2*	*4*	0.5	*2*	*0*	*2*	0	*3*	*1*	*2*	0.5
	01_25_07	CE carbohydrate esterase	*3*	*1*	*2*	0.5	*0*	*0*	*0*	0	*3*	*3*	*0*	n.a.
transcription	11_02_03_04	C2H2 transcriptional regulator	*5*	*2*	*3*	0.7	*5*	*0*	*5*	0	*9*	*3*	*6*	0.5
	11_02_03_04	transcription factor	*8*	*1*	*7*	0.1	*0*	*0*	*0*	n.a.	*0*	*0*	*0*	n.a.
	11_02_03_04	Zn2Cys6 transcriptional regulator	*11*	*3*	*8*	0.4	*3*	*0*	*3*	0	*5*	*2*	*3*	0.7
protein fate	14_07_04	GCN5-N-acetyltransferase	*6*	*3*	*3*	1	*2*	*1*	*1*	1	*1*	*1*	*0*	n.a.
	14_07_09	SAM-dependent methyltransferase	*4*	*4*	*0*	n.a.	*3*	*0*	*3*	0	*3*	*2*	*1*	2
protein with binding functions	16_01	ankyrin	*2*	*1*	*0*	n.a.	*2*	*0*	*2*	0	*0*	*0*	*0*	n.a.
	16_21	cytochrome P450 subfamily	*8*	*6*	*2*	3	*9*	*0*	*9*	0	*5*	*4*	*1*	4
	16_21	FAD-dependent monooxygenase	*11*	*8*	*3*	2.7	*3*	*2*	*1*	2	*2*	*0*	*2*	0
	16_21_07	GMC oxidoreductase	*2*	*2*	*0*	n.a.	*2*	*0*	*2*	0	*1*	*0*	*1*	0
	16_21_07	short chain dehydrogenase/reductase	*8*	*5*	*3*	1.7	*11*	*0*	*11*	0	*6*	*0*	*6*	0
cellular transport	20_01_01	oligopeptide transporter	*8*	*3*	*5*	0.6	*1*	*1*	*0*	n.a.	*3*	*1*	*2*	0.5
	20_01_01_01_01	iron permease	*2*	*2*	*0*	n.a.	*0*	*0*	*0*	n.a.	*0*	*0*	*0*	n.a.
	20_01_01_07	inorganic phosphate transporter	*4*	*4*	*0*	n.a.	*1*	*1*	*0*	n.a.	*1*	*1*	*0*	n.a.
	20_01_03	monocarboxylate transporter	*3*	*3*	*0*	n.a.	*0*	*0*	*0*	n.a.	*0*	*0*	*0*	n.a.
	20_01_07	amino acid permease	*5*	*1*	*4*	0.3	*3*	*3*	*0*	n.a.	*4*	*3*	*1*	3
	20_03	MFS (major facilitator superfamily) (12 TMH)	*51*	*15*	*36*	0.4	*7*	*4*	*3*	1.3	*17*	*9*	*8*	1.1
signal transduction mechanism	30_05_02_24	GPCR, G-protein coupled receptor	*6*	*1*	*5*	0.2	*0*	*0*	*0*	n.a.	*2*	*0*	*2*	0
	30_05_02_24	PTH11-type GPCR	*11*	*9*	*2*	4.5	*2*	*0*	*2*	0	*2*	*1*	*1*	1
cell rescue and defense	32_01_05	chaperone/heat shock protein	*0*	*0*	*0*	n.a.	*5*	*5*	*0*	n.a.	*4*	*0*	*4*	0
	32_07	AAA + −type ATPase	*4*	*2*	*2*	1	*8*	*0*	*8*	0	*2*	*1*	*1*	1
	32_07_05	PDR-type multidrug transporter	*3*	*3*	*1*	3	*0*	*0*	*0*	n.a.	*2*	*1*	*1*	1
	32_07_05	ABC transporter	*0*	*0*	*0*	n.a.	*2*	*0*	*2*	0	*2*	*0*	*2*	0
	32_07_05	MDR (multidrug resistance-associated protein)	*0*	*0*	*0*	n.a.	*2*	*0*	*2*	0	*1*	*0*	*1*	0
	32_07_07_03	glutathione S-transferase	*3*	*3*	*0*	n.a.	*4*	*0*	*4*	0	*2*	*0*	*2*	0
subcellular localization	70_01	cell wall protein	*5*	*0*	*5*	0	*2*	*0*	*2*	0	*4*	*2*	*2*	1
	70_27	SSCR (small secreted cysteine rich protein)	*8*	*8*	*0*	n.a.	*6*	*0*	*6*	0	*0*	*0*	*0*	n.a.
cell fate	40	HET protein	*2*	*2*	*0*	n.a.	*0*	*0*	*0*	n.a.	*1*	*0*	*1*	0

*ratio values cannot be given when denominator is zero; n.a. means not applicable.

**Figure 4:**
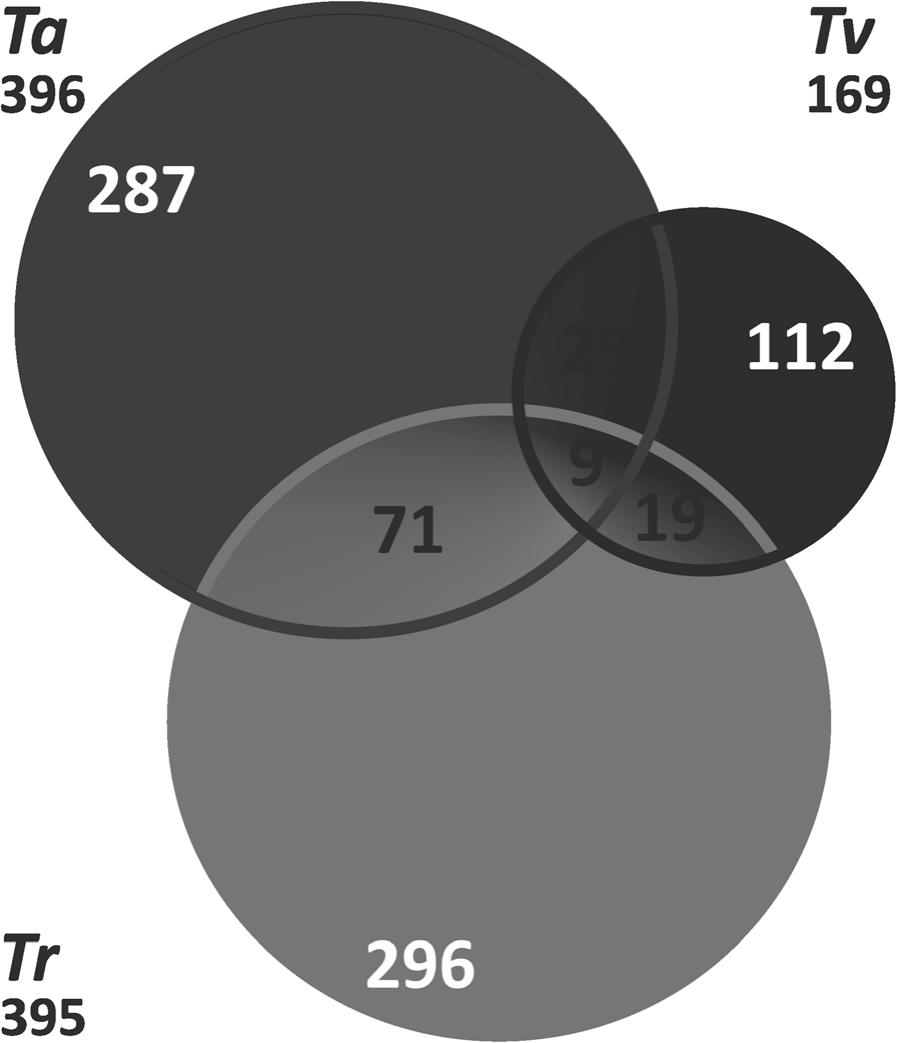
**The Venn diagram showing the number of orthologous genes of *T. reesei*, *T. atroviride *and *T. virens *that are involved in mycoparasitic response against *Rhizoctonia solani *(at all stages of confrontation).** The total number of orthologous genes per species is given below the respective species name.

Interestingly, the majority of significantly up-regulated genes were represented by genes for which orthologues in other *Trichoderma* species are present (Figure 4). In addition, 88 of these genes were shared only between *Tv* and *Ta*. Thus 93.2, 74.4 and 84.8% of all genes that were significantly expressed in either *Tr, Ta* or *Tv*, respectively, were orthologues or at least conserved in *Ta* and *Tv*. In contrast, among those most of genes involved in mycoparasitic response were only found in one species. Only nine orthologuous genes were up-regulated in all three species (Figure 4) when confronted with *R. solani*, and 29 genes were significantly expressed only in the two mycoparasitic species *Ta* and *Tv*; they comprised 16 unknown, three putative MFS transporters, two orphan and two unknown transcriptional activators genes.

The most significant differences were evident at the pre-contact stage (ANOVA, p < 0.001): in *Tr,* the majority of differentially regulated genes were up-regulated, whereas in *Tv* and in *Ta* genes in this stage were mostly down-regulated and showed only a small response (Figure 3). At contact with hyphal tips of *R. solani*, *Tr* and *Ta* displayed only a low transcriptomic response, whereas *Tv* showed significant down-regulation of most of its genes (ANOVA, p < 0.001). During overgrowth of the prey, expression of most of genes increased in *Ta* and *Tv*, but were down-regulated in *Tr.* However, in general transcriptional response in this stage was significantly different between *Ta* and the other two species (ANOVA, p < 0.001).

To confirm the microarray results, quantitative Real-Time-PCR (qPCR) was performed on a subset of the genes from *Tv* and *Ta*. They were chosen from gene families that showed abundant up-regulation during confrontation with *R. solani*, such as GH16 glucanases, proteases, PKS and SSCPs (see below). Expression of these genes correlated well with the data of the microarray (Additional file 1: Table S4). We therefore conclude that the results of tiling microarray analysis indeed reflect differences in the function of genes involved in mycoparasitism.

**Figure 5:**
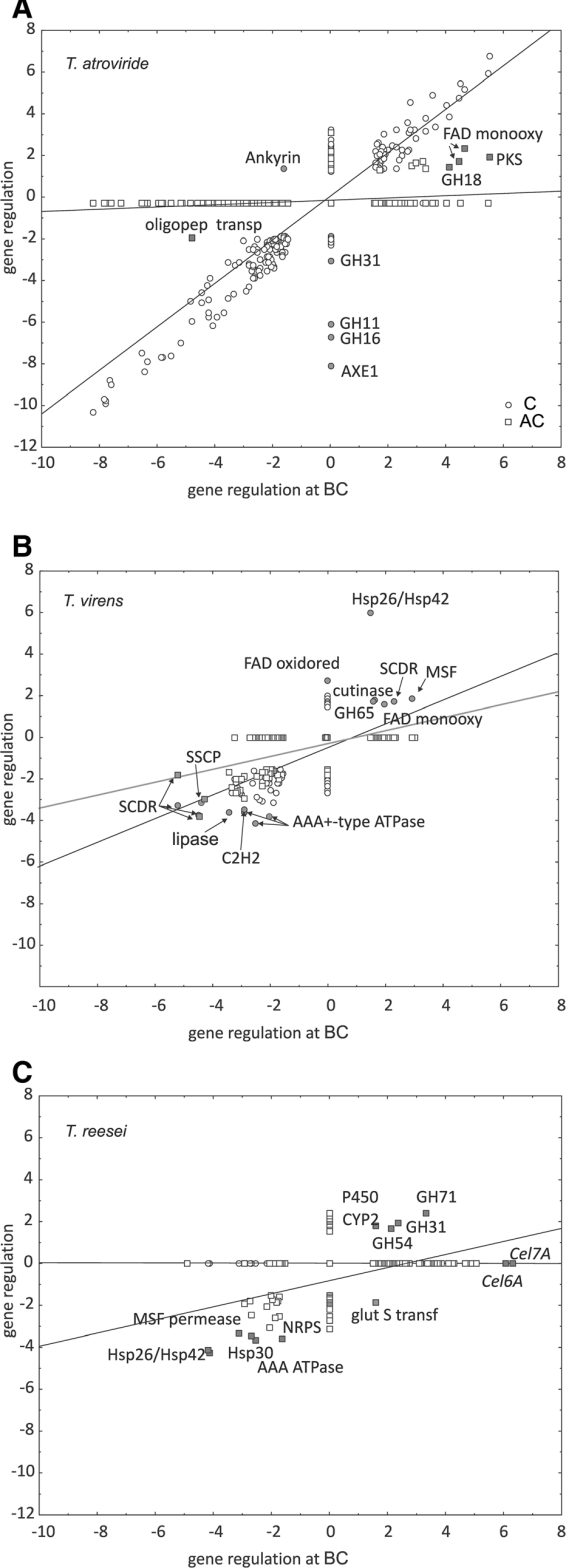
**Scatter plots of all regulated genes in each stage for (A) *T. atroviride*, (B) *T. virens *and (C) *T. reesei*, respectively.** The log_2_ (ratio) regulation for the genes at contact (C, circles) and after the contact (AC, squares) stages are plotted against the genes regulated prior the contact (BC). The genes, for which a stage-specific difference was found, are indicated by their functional names.

The enrichment for the major functional categories (FunCat) was assessed for each category (Table 2) separately. To reveal the common (shared by all three species) and species-specific mechanisms acting at every stage of interaction between *Trichoderma* and *R. solani* we performed the statistical analysis of the respective FunCats. In general, all three species showed significantly different FunCat profiles indicating the presence of alternative interaction strategies (MANOVA; p < 0.05). Shifts in gene expression between BC, C and AC stages of the interaction were also strongly species-specific (Figure 5): only a minor portion of genes became down-regulated in C compared to BC and at AC compared to C (ANOVA < p < 0.001). However the massive differences were observed in *Ta* when a large group of genes became up-regulated at AC compared to BC and C (Figure 5A).

Statistical analysis of the global comparison of FunCat categories for the three stages for *Tr, Tv* and *Ta*, revealed that most categories broadly scattered both in the up- and down-regulated areas (Table 2). However, some specific features were observed: in *Tr*, genes involved in metabolism and transport were all up-regulated and the latter also in *Tv*; expression of proteins with binding function was increased in *Ta* during the contact phase and proteins involved in self-defense decreased in *Tr* during overgrowth. It is also apparent that during overgrowth regulation of all genes was much more comparable (as indicated by less scattering of values), and only few categories also included up-regulated genes.

### Genes up-regulated in interaction with *R. solani*

A comparison of the protein families [Pfam, http://pfam.sanger.ac.uk/] assigned for up-regulated genes in the above analysis revealed very little common responses among the three *Trichoderma* spp., and also between the two opportunistic and strong mycoparasitic species. In fact only a few gene families shared a common trend in all three *Trichoderma* spp. (proteases, heat shock proteins, cytochrome C peroxidase, proline oxidase, ER-bound glutathione-S-transferases, ABC efflux transporters, the pleiotropic drug resistance (PDR) transporters, multidrug resistance MDR-type transporters and nitrilases), indicating that stress response connected with detoxification of potentially hazardous metabolites is a general reaction of *Trichoderma* during antagonism (see the Discussion below).

In addition to the genes named above, all three *Trichoderma* spp. had distinct specific responses. Thus, in addition to expression of the highest number of several protease families (dominated by subtilisin-like and aspartyl proteases), *Ta* increased the transcription of oligopeptide transporters, C-type lectins, small secreted cysteine-rich proteins (SSCPs), PTH11-receptors and ß-glucanases of the GH16 family (Table 2; Additional file 1: Tables S1-S3). In contrast, *Tv* mainly increased the expression of genes for gliotoxin biosynthesis including the genes for its precursors, and genes encoding heat shock proteins. The minor additional responses in *Tv* included increased up-regulation of chaperone proteins, and several dehydrogenases and monooxygenases and significant down-regulation of AAA + −type ATPases and transcriptional regulators. However, it was conspicuous that both strongly opportunistic and mycoparasitic species expressed all these genes already at a stage before hyphal contact with *R. solani,* and were not expressed anymore after the contact with the prey (Figure 5B).

*Ta* also largely relied on antibiosis: one polyketide synthase (PKS) of the reducing (lovastatin/citrinin) clade I (Triat2:134224; [17]) was expressed at all stages of interaction, and peaked at C. A second PKS gene (Triat2:85006; [17]) from the reducing clade I was expressed only at contact but not at BC and AC. Interestingly, *Ta* also expressed genes encoding two KP4-like killer-toxins that have so far not been described for filamentous fungi. In addition, the up-regulation of genes encoding macrophomate synthases, isoflavone reductases and pyoverdin dioxygenases was notable. These genes may be involved in the formation of yet unknown secondary metabolites of *Trichoderma*. Finally, a gene encoding a lipoxygenase (Triat2:33350), which is absent in *Tr* and *Tv,* was up-regulated at C stage. It may be involved in the biosynthesis of γ-pentylpyrone (see details in the Discussion).

In contrast, the transcriptome of *Tr* before contact revealed unique response to the presence of *R. solani*. It showed a massive up-regulation of cellulolytic and hemicellulolytic CAZymes, and ribosomal proteins (Table 2). Furthermore, several transporter proteins (MSF permeases, inorganic phosphate, oligopeptide and amino acid transporters) as well one PKS (Trire2:60118) were up-regulated at this stage. Interestingly, *Tr* also up-regulated subtilisin proteases but none of the aspartyl proteases that were active in *Ta*. At C stage *Tr* significantly reduced the total gene expression, and only genes encoding a multidrug transporter, a stress-responsive protein (RDS1) and another PKS were induced and may indicate defense reaction rather than an attack. The AC stage was characterized by an increased activity of the genes encoding ribosomal proteins, while genes for cellulolytic and hemicellulolytic CAZymes were not expressed anymore.

### Genes down-regulated in interaction with *R. solani*

Some gene families were consistently down-regulated in all three species during confrontation with *R. solani*, particularly already at BC stage. These comprised short-chain dehydrogenases/reductases, transcriptional regulators of the fungal-specific Zn(2)Cys(6) family, non-ribosomal peptide synthases, and AAA + −domain proteases.

The only species-specific response was detected for *Ta,* which showed the opposite expression pattern of genes involved in polysaccharide and lipid hydrolysis and solute uptake to *Tr*, as it strongly down-regulated all these genes already prior the contact (BC).

### Genomic distribution of genes involved in mycoparasitism

We also investigated whether the genes expressed during interaction with *R. solani* would be clustered in the genome. Our approach was based on the consideration that under random distribution of transcribed genes, we should (on the average) find them once in batches of 18, 42 and 22 subsequent genes in *Ta*, *Tv* and *Tr*, respectively. These values arise by division of the total number of genes in the *Ta, Tv* and *Tr* (11865, 12518, and 9143, respectively) by the number of genes significantly expressed by them in this study (651, 303 and 424, respectively). To detect significant deviations from these expectations, we manually screened the three genomes for genes that occurred within the vicinity of each other at an at least 3-fold lower value than the above calculated average distributions, as was also done by Seiboth, Aghcheh et al. [18]. We detected that the distribution of about one third of the genes (36.9, 34.7 and 37.2% in *Ta, Tv* and *Tr*, respectively) was not random (Pearson coefficient >0.25) (Table 3). About a third of them were located at the ends of the respective scaffolds implying that they are situated either at the ends of chromosomes or nearby repetitive regions.

**Table 3:** **Clusters of nonrandomly distributed genes in *T. atroviride, T. virens *and *T. reesei *that are involved in mycoparasitism**

**Scaffold**	***T. atroviride***		***T. virens***		***T. reesei***	
	Gene area*	Density**	Gene area*	Density**	Gene area*	Density**
**1**	44-47	3/4	583-618	6/36	62-68	3/7
	69-87	7/19	974-990	4/17	129-135	4/7
	1111-1119	4/9			439-452	4/14
	1331-1340	3/10			632-639	3/8
	1717-1726	3/10			734-753	5/20
**2**	85-93	3/9	3-27	7/25	43-55	4/13
	306-313	3/8	134-148	3/15		
	649-663	6/15	253-274	4/22		
	936-942	3/7	665-695	4/31		
	998-1000	3/3				
	1031-1039	3/9				
	1039-1054	6/16				
**3**	140-143	4/4	788-792	4/5	4-27	5/24
	189-201	10/13			333-342	3/10
	691-698	3/8			406-411	4/6
	771-773	3/3				
**4**	37-45	4/9	20-24	4/5	34-45	3/12
	340-349	3/10	242-255	3/14	333-339	3/7
	593-614	6/20	327-346	3/20		
**5**	12 - 24	7/13	252-262	3/11	209-226	4/14
	97-104	3/8			235-248	4/14
	286-302	5/17			448-463	5/16
	389-412	8/22				
	425-427	3/3				
**6**	264-268	4/5	2-21	3/20	24-51	6/28
	354-357	3/4			128-139	3/12
	461-483	7/23				
**7**	178-187	4/10	6-9	3/4	59-72	5/14
	404-408	3/5			286-292	3/7
	484-509	8/26				
	524-532	5/9				
	546-559	4/14				
**8**	117-129	6/13			276-290	3/15
	155-165	6/11				
	185-189	3/5				
**9**	173-188	6/16			26-44	4/19
	198-212	4/15				
	489-498	4/10				
**10**			242-262	3/21	74-83	3/10
					185-187	3/3
					262-272	3/11
					316-344	5/29
**11**	399-411	5/13				
	462-477	9/16				
**12**	386-391	3/6			7-11	3/5
	466-468	3/3			160-174	3/15
**13**	77-85	3/9	277-281	3/5		
	132-139	3/8				
	275-281	3/7				
			219-229	3/11	1-14	3/15
**15**	20-25	4/6				
	169-174	3/6				
**16**	55-61	3/7				
**17**	78-85	3/8			59-86	6/28
	165-175	4/11				
**18**	45-62	6/18	1-7	5/7		
	135-138	3/4				
**19**	4 of 12	3/9	107-113	4/7	32-37	3/6
	68-82	6/15				
	141-155	4/15				
**20**	2-11	5/10			94/100	3/7
**21**			21-41	3/21	116-120	4/5
			100-109	3/10		
**22**			101-114	4/15	80-109	7/30
**24**					29-41	4/13
**26**			53-63	4/11		
**27**					40-56	4/17
**28**			32-51	3/20	68-74	3/7
					98-122	5/25
**29**					68-79	3/12
					99-113	6/13
**32**					55-62	3/8
**34**			3-13	10/11	26-30	3/5
**35**			32-51	4/20		
**45**					7-20	3/14
**54**			1-8	5/8		

* indicates the area on the scaffold in which the cluster was identified, given by the numbers of the first and the last genes on the scaffold. ** the ratio of significantly up- or down-regulated genes to the total number of genes in the cluster.

## Discussion

Although a wide range of *Trichoderma* spp. have been described as mycoparasites [1], detailed studies on the molecular physiology of this trait have been performed mostly with *T. harzianum* CECT 2413 or the *T. atroviride* strains P1 and IMI 206040 [2,3,19]. Based on these studies, it is commonly believed that the expression of cell wall lytic enzymes (particularly chitinases but also ß-glucanases and proteases) and secondary metabolites are the major determinants for success in this process. The present comparison of the two mycoparasites *T. atroviride* and *T. virens* with *T. reesei*, which was unable to efficiently besiege *R. solani*, changes this view in two important aspects: first, the role of induction of hydrolytic enzymes and secondary metabolites must be revisited; and second, the two mycoparasites display completely different strategies to antagonize their host/prey.

The latter claim is nicely reflected by the findings that – although the majority of significantly expressed genes in the three *Trichoderma* spp. belong to the gene inventory present in all of them – most of these genes are specific for only in one of the species. This implies that rather the regulation of gene expression than the availability of specific “mycoparasitic” genes is the key for successful antagonism. The molecular basis for this has not yet been studied: it could be the enhanced number of transcription factors in *Ta* and *Tv*[4] which may have resulted in a more refined transcription pattern of genes related to mycoparasitism. The other reason could be the occurrence of many of these genes in nonsyntenic regions of the genome (at the ends of chromosomes or nearby repetitive regions; *vide supra*), which could have changed the transcriptional environment of these genes.

We have previously reported that *T. atroviride* is able to sense the presence of its host from a distance [20]. This ability to sense the other fungus seems to be a property of all *Trichoderma* species, as all three show a significant and specific expression of a number of genes already before contact. However, the types of genes expressed distinguished *T. atroviride* and *T. virens* from *T. reesei*: while the former two species express a number of genes for which a function in antagonism can be interpreted, *T. reesei* mainly expresses genes for nutrient acquisition. Interestingly, the expression of the same cellulase and hemicellulase genes was strongly down-regulated in *T. atroviride* and highly induced in *T. reesei, T. virens* remained unaffected. One interpretation of this observation would be that – upon sensing a potential prey – *T. atroviride* changes gene expression towards an attack, whereas *T. reesei* attempts to compete with the other fungus by faster acquisition of nutrients. Likewise, it is possible that the sensing of a basidiomycete fungus signals the availability of pre-degraded wood to *T. reesei*, in accordance with the model that *T. reesei* became an efficient saprotroph on dead wood by following wood-degrading fungi into their habitat [1,20,21]. This implies that components from the host/prey are able to stimulate this process, and the role of the general cellulase regulator XYR1 [22] therefore warrants examination. The fact that cellulase gene expression came to an immediate stop when *T. reesei* arrived at physical contact with *R. solani* does not contradict this explanation, because cellulases and hemicellulases are secreted into the medium where they have a half-life of 40 and more hours. Thus the cellulases secreted by *T. reesei* before contact may suffice for nutrient acquisition throughout the whole process of interaction observed.

**Figure 6:**
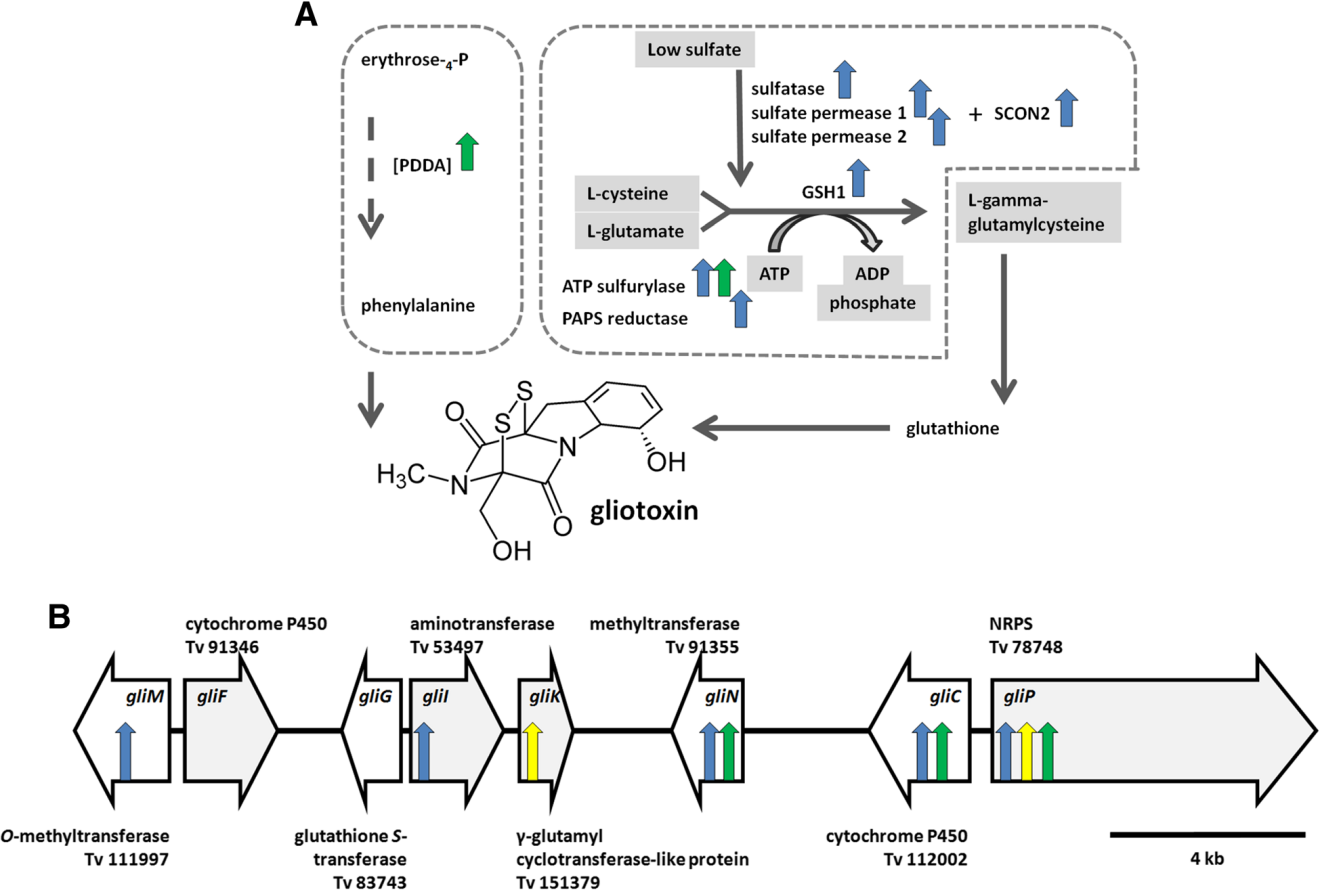
**Gliotoxin and gliotoxin precursor biosynthesis in *T. virens *during mycoparasitism with *R. solani*. A**) Only enzymes, whose genes are up-regulated are shown, marked as blue, yellow and green arrows for gene up-regulation before contact (BC), at the contact (C) and after the contact (AC), respectively. **B**) The arrows show the gliotoxin gene cluster including gene nomenclature, protein numbers and their regulation (blue, yellow and green arrows marking up-regulation BC, at C and AC, respectively) during mycoparasitic reaction with *R. solani*.

The two strong mycoparasitic species also differed in the tools that they used for combating and killing *R. solani*. *T. virens* straightforwardly headed for predation by poisoning *R. solani* with gliotoxin. Earlier studies [23-27] have already suggested that gliotoxin formation is the main antifungal principle of a subset of *T. virens* strains, so called “Q-strains” (such as Gv29-8). Our data are consistent with this claim, but it must be noted that they may not apply to “P-strains” as they do not produce gliotoxin [25,26]. In our experiments it was evident from the up-regulation of almost all genes required for gliotoxin biosynthesis, i.e. a two module non-ribosomal peptide synthetase (*gliP*); thioredoxin reductase (*gliT*); O-methyl transferase (*gliM*); a methyl transferase with unknown specificity (*gliN*); glutathione *S*-transferase (*gliG*); cytochrome P450 monooxygenases (*gliC*); amino cyclopropane carboxylate synthase (*gliI*); and a dipeptidase (*gliJ*). This coordinated expression is also supported by the genomic clustering of these genes (Figure 6). However, it was of interest to note that also genes involved in the provision of the precursor of gliotoxin, L-phenylalanine, and of the glutathione required for the formation of the central disulfide bond were induced during one or more of the three stages. Provision of sulfur for cysteine and subsequently glutathione biosynthesis appeared to be an essential requirement already before contact, as indicated by the up-regulation of two sulfate permeases, one sulfatase, the cysteine biosynthesis genes ATP-sulfurylase and PAPS reductase, and of SCON2, an ubiquitin-ligase involved in regulating sulfur metabolism under conditions of low sulfate supply [28]. Most of these genes were up-regulated only before contact, suggesting that *T. virens* could later fulfill the sulfur requirement by feeding on *R. solani*. In contrast, a shortage in L-phenylalanine appears to take place only during overgrowth as illustrated by the increased expression of 3-deoxy-D-arabino-heptulosonate-7-phosphate synthase, the first enzyme involved in the biosynthesis of aromatic amino acids, and also the regulatory target for this pathway [29].

It is interesting to note that the genes of the gliotoxin gene cluster that are present in *Tr*[23] were not at all expressed during confrontation with *R. solani*, whereas the gliotoxin cluster is absent in *Ta*[23].

*T. atroviride*, in contrast, appeared to follow a strategy that involved antibiosis as well as the action of hydrolytic enzymes indicating a rather parasitic interaction that does not directly aims to kill the host. With respect to the latter, *T. atroviride* up-regulated almost only ß-1,3/1,4-D-glucanases of the GH16 family, of which some also contained a carbohydrate-binding domain of the lectin superfamily (SCOP link 49899). Unfortunately their properties have not been studied in sufficient detail to offer an interpretation why *T. atroviride* overexpressed just this battery of ß-glucanases and not one of the several others that are present in its genome [4].

Proteases, on the other hand, were up-regulated in all three species, although only the aspartyl proteases were consistently up-regulated in the two strong mycoparasites and down-regulated in *T. reesei*. This confirms our earlier conclusion [30] that the proteases play an important role in mycoparasitism.

Up-regulation of genes for secondary metabolite production in *T. atroviride* most conspicuously comprised two PKS genes of the reducing (lovastatin/citrinin-like) clade I [17]. One of them (Triat1:134224) was in fact one of the most up-regulated genes before and at contact with *R. solani*. However, there is indirect evidence for yet more secondary metabolite formation: *T. atroviride* has been reported to produce α–pentyl-pyrone, a volatile component with antifungal activity that is not formed by *T. reesei* and *T. virens*[31]. Its biosynthesis has been suggested to occur via linoleic acid, in analogy to jasmon synthesis by plants in which the necessary hydroxyl group originates from an oxidation by lipoxygenase [32]. A lipoxygenase gene was indeed present in *T. atroviride* (protein ID 33350) but not the two other species, and was significantly up-regulated in *T. atroviride* during contact with *R. solani*. In addition, the up-regulated genes putatively encoding macrophomate synthase, pyoverdin dioxygenase and of isoflavone reductase may be involved in the biosynthesis of so far unknown secondary metabolites. Two PKS (Trire2:60118, Trire2:82208; the first of the same type as the two of *T. atroviride*, and the second belonging to the nonreducing PKS clade I), were also up-regulated in *T. reesei* before and at contact respectively. However, no other genes with potential relationship to secondary metabolism were observed in *T. reesei*. Interestingly, genes encoding nonribosomal peptide synthases (NRPS) – including the two encoding peptaibol synthases, which have been emphasized as important antifungal components from *Trichoderma*[33] – were found to be down-regulated in all three species. Similar findings have been reported recently for *T. atroviride* by Reithner et al. [5].

Another notable finding with *T. atroviride* was the up-regulation of several genes encoding members of GPCR PTH11 class [34], of lectins and of small secreted cysteine rich proteins. PTH11 encoding genes were also among those up-regulated in the mycoparasite *Coniothyrium minitans* during colonization of *Sclerotinia sclerotiorum*[35], thus rendering them candidates for a function in mycoparasitism. Interestingly, a PTH11 receptor of *Botrytis cinerea* is not involved in plant pathogenicity but regulates the expression of a glutathione-S-transferase gene [36]. The lectins also included a gene encoding cyanovirin, a mannose-binding lectin [37]. Lectins have been suggested to be involved in coiling of *Trichoderma* around hyphae of the fungi they attack [38]. SSCPs are abundant in *Trichoderma*[4] and the high expression of several of them in *T. atroviride* suggests a potential role in mycoparasitism. They are involved in the mutualistic behavior of the ectomycorrhizal symbiont of plants *Laccaria bicolor* and are active in some plant pathogenic fungi [39].

Only a few gene families shared a common trend: all three *Trichoderma* spp. also displayed an oxidative response in confrontation with *R. solani* (expression of heat shock proteins, cytochrome C peroxidase, proline oxidase, and ER-bound glutathione-S-transferases). Likewise, genes for detoxification processes (ABC efflux transporters, the pleiotropic drug resistance (PDR) transporters and the multidrug resistance MDR-type transporters) were induced. *R. solani* uses radical oxygen species as signaling molecules during sclerotia formation [40], and excretes antifungal components [41], both of which could act in our experiments and may have elicited this response. An ABC-transporter from *T. atroviride* (TAABC2) is involved in biocontrol of *R. solani*[42].

A cyanide hydratase gene with high similarity to corresponding proteins from other ascomycetes was one of the most strongly induced genes in all three *Trichoderma* spp. at all stages of the interaction. The fungal cyanide hydratases form a functionally specialized subset of the nitrilases which catalyze the hydrolysis of cyanide to formamide [43]. One could argue that this nitrilase serves defend against cyanide produced by *R. solani*, but this has not yet been proven so far. On the other hand, bacteria like *Pseudomonas* spp. [44] produce cyanide. Since this gene was expressed by all three *Trichoderma* spp., its expression may be a general response of *Trichoderma* towards the presence of any host, but this clearly needs further investigations.

A significant portion of the genes responding to the presence of *R. solani*, were non-randomly distributed in the genome of all three *Trichoderma* spp. In ascomycetes clustering is primarily known for genes involved in secondary metabolite synthesis [45]. In *T. reesei* the genes encoding cellulases, hemicellulases and other proteins of the CAZome also occur in clusters [46], and we have recently shown that the genes regulated during conidiation also are clustered in the genome [47]. In *Fusarium graminearum* (teleomorph: *Gibberella zeae*) [48] and *Neurospora crassa* (anamorph: *Chrysonilia crassa*) [49], clustering occurs in subtelomeric regions and contains particularly fast evolving genes, such encoding secreted proteins and orphan genes related to ecological success of an organism. In this context, it is reasonable that the genes related to mycoparasitism are clustered in organisms that have specialized on this trait. In *Aspergillus* spp., expression of genes from such clusters is controlled by the putative methyltransferase LaeA [50], a component of the Velvet protein complex [51]. In this regards, we have recently shown that cellulase formation and conidiation in *T. reesei* are in fact regulated by the orthologue LAE1 [18]. Another component of the Velvet protein complex, VEL1 (VeA) controls mycoparasitism in *T. virens*[52]. We were recently able to show that LAE1 indeed is a regulator of mycoparasitism in *T. atroviride* (R.K. Aghcheh, I.S. Druzhinina and C.P. Kubicek, unpublished data).

## Conclusions

The present comparison of the two opportunistic and strongly mycoparasitic species *T. atroviride* and *T. virens* with *T. reesei*, which is unable to besiege *R. solani*, reveals different responses of *Trichoderma* spp. to the presence of another fungus. It also demonstrates that there is no common mechanism by which a mycoparasite attacks and kills its host, but that alternative strategies are used. Finally, the observation of a nonrandom distribution of the transcribed genes suggests the possible involvement of epigenetic regulation of mycoparasitism.

## Methods

### Comparative transcriptomics analysis

For mycoparasitism confrontation assays *T. virens* Gv29-8, *T. atroviride* IMI 206040 and *T. reesei* QM 6a were grown on potato dextrose agar plates (BD Dicfo, Franklin Lakes, NJ, USA), covered with cellophane, at 25°C and 12 hours cyclic illumination and harvested when the mycelia were ca. 5 mm apart, at contact of the mycelia and after *Trichoderma* had overgrown the host fungus *Rhizoctonia solani* by ca. 5 mm. As control, the respective strain of *Trichoderma* was confronted with itself and harvested at contact. Peripheral hyphal zones from each confrontation stage were sampled and shock frozen in liquid nitrogen. Mycelia were ground to a fine powder under liquid nitrogen and total RNA was isolated using the guanidinium thiocyanate method [53]. For cDNA synthesis, RNA was treated with DNase I (Fermentas, Burlington, Canada) and purified with the RNeasy MiniElute Cleanup Kit (Qiagen, Valencia, CA, USA). 5 μg RNA/reaction were reverse transcribed using the SuperScript™ III Reverse Transcriptase (Invitrogen, Carlsbad, CA, USA) and a mixture of provided random hexamer primer and oligo(dT) primer.

We designed *T. virens*, *T. atroviride* and *T. reesei* tiling array with 60mer oligonucleotides (oligo length) each 93 bp apart (oligo distance), using the unmasked fasta file of the genomes of *T. atroviride* [http://genome.jgi.doe.gov/Triat2/Triat2.info.html], *T. vir*ens [http://genome.jgi-psf.org/Trive1/Trive1.home.html] and *T. reesei* [http://genome.jgi-psf.org/Trire2/Trire2.home.html]. The design was done with Teolenn [54] using a maximum prefix length match set to 30 for the uniqueness calculation made by genome tools. Complexity is evaluated using the masked genome by counting the number of masked bases for each probe. T_m_ values are calculated using the nearest neighbor thermodynamic model. No filters were applied after probe parameter calculations. In order to obtain the probe quality score, the calculated parameters were weighted as follows: 0.4 for T_m_, 0.3 for uniqueness, 0.2 for GC content, and 0.1 for complexity. To get final oligonucleotide scores, a weighting of 0.75 was assigned to quality scores, and a weighting of 0.25 was assigned to position scores. Teolenn software designed 415373, 387105 and 359089 final probes respectively for *T. atroviride*, *T. virens*, and *T. reesei*. Oligonucleotides were loaded on Agilent eArray software and 2 × 400 k microarrays were obtained from Agilent. The microarray data and related protocols are available at the GEO web site [http://www.ncbi.nlm.nih.gov/geo/] under accession number GSE23438. Briefly, the RNAs of two independent biological replicates for each condition (which in turn consisted of pooled RNA preparations from at least three separate cultivations) were reverse-transcribed and labeled with Cy3 or Cy5 dye using the indirect labeling procedure. Dye bias was eliminated using dye switch labeling protocol. We then hybridized 1.5 μg of labeled cDNA with the 2x400k DNA chip (Agilent). The array was read using an Agilent G2505C DNA microarray scanner and the TIFF images extracted with the Agilent Feature Extraction software (version 10.5.1.1) using the 20bit coding ability. Data pre-treatment was applied on each result file to discard flagged spots by Feature Extraction software. The data were normalized without background subtraction by the global Lowess method performed with the Goulphar software [55]. For each experimental condition, the two file results were merged together. For each probe the hybridization ratio was linked to genome annotation coming from the DOE JGI website for a respective genome. The final ratio for each transcript was obtained by averaging the detected hybridization values from all probes located inside the coding sequence on the matching strand. Transcripts with no or only one probe marked as detected were discarded from further analysis. Finally we kept only transcript with a final hybridization ratio greater than log_2_ (ratio) = 1.5 or lower than log_2_ (ratio) = −1.5. The list of statistically significant differentially expressed genes was obtained by applying the linear modeling approach implemented in lmFit package for Python and the empirical Bayes statistics implemented by eBayes from the limma R package (http://bioinf.wehi.edu.au/limma/; [16]).

### Gene identification

The genomes of *T. reesei* QM6a, *T. atroviride* IMI 206040 and *T. virens* Gv29-8 have been annotated [4] and were used as a source for identification of the genes found in this study. A gene was considered to encode a homologue of an already identified protein if it had the Expect value (E) in blastp of < e^-100^ with the next Sordariomycetes member. All unknown and orphan (unique) proteins were subjected to blastp search (May, 2012) to check whether any of them has been identified for another fungus since the *Trichoderma* annotations. Orphan proteins were defined as such that did not occur in any other Pezizomycotina with an E-value > e^-20^. All other proteins, for which no function could be predicted, were termed as unknown.

### Statistical data evaluation

Statistical analyses of microarray data were performed using Statistica 6.1 (StatSoft, Inc., Tulsa, OK, USA). For this purpose the cumulative matrix has been assembled (Additional file 1: Table S1-S3). In this matrix every gene was considered as a case and the following grouping variable (predictors) were integrated: protein ID, gene annotation, species, protein family (Pfam), gene regulation (up-regulated/down-regulated), fold gene regulation prior, at and after the contact, and functional category (FunCat). Data were explored by means of descriptive statistics (mean values and standard deviations were calculated for each predictor). Hypotheses were tested using one-way main effects or factorial ANOVA analyses with post-hoc comparisons with Tukey honest significant difference tests (Tukey HSD).

### Mycoparasitic potential

The antagonistic potential was evaluated for each species based on dual confrontation plate assay carried out as described for the RNA extraction, except the plate were not covered with cellophane. The evaluation of the plates was carried out after 10 days of incubation (see Figure 1) and the growth of *Trichoderma* against itself was set up as a zero inhibition rate. The growth inhibition of *R. solani* was calculated implying that the distance between the plugs of both fungi is 100%. The inhibition of growth was calculated as a percentage of *Trichoderma* growth subtracted for a correction of the growth against itself. Overgrowth of the prey was calculated similarly using the distance between the *R. solani* plug and the confrontation zone with *Trichoderma* as 100%, and the growth of *Trichoderma* over the *R. solani* mycelium was then calculated as a percentage of overgrowth.

### Real Time PCR quantification of transcripts

*T. atroviride* and *T. virens* were cultivated and harvested as described for the microarrays. DNase treated (DNase I, RNase free; Fermentas) RNA (5 μg) was reverse transcribed with the RevertAid™ First Strand cDNA Kit (Fermentas) according to the manufacturer’s protocol with a combination of the provided oligo(dT) and random hexamer primers.

All real-time PCR experiments were performed on a Bio-Rad (Hercules, CA) iCycler IQ. For the reaction the IQ SYBR Green Supermix (Bio-Rad, Hercules, CA) was prepared for 25 μl assays with standard MgCl_2_ concentration (3 mM) and a final primer concentration of 100 nM each. Primers used are given in Additional file 1: Table S4. The amplification protocol consisted of an initial denaturation step (3 min at 95°C) followed by 40 cycles of denaturation (15 sec at 95°C), annealing (20 sec at 52 and 52.5°C for *T. virens* and *T. atroviride* respectively) and extension (72°C for 10 sec for both species). *Tef1* gene which maintained constant over all conditions (± 20 relative %) was used as the normalizer. Determination of the PCR efficiency was performed using triplicate reactions from a dilution series of cDNA (1, 0.1, 10^-2^ and 10^-3^). Amplification efficiency was then calculated from the given slopes in the IQ5 Optical system Softwarev2.0. The qPCR were performed with the cDNA of 5 pooled biological replicates for each species and condition. Expression ratios were calculated using Livak test model [56] and are given in the Additional file 1: Table S4. Zero values for qPCR indicate the expression levels between |log_2_ (ratio)| > 1.5 as these were not treated as significantly regulated in the microarray analysis.

## Competing interests

The authors declare that they have no competing interests.

## Authors’ contributions

LA performed the experiments with *Tr* and *Ta*, carried out qPCR, did the statistical data evaluation, prepared some figures and supplementary materials and participated in ms writing. SLC and FC made microarrays. SG made experiments with *Tv*. VS participated in the design of the study and gene annotation. CPK and ISD designed the study, evaluated the data and wrote the manuscript. ISD prepared some figures and supplementary materials. All authors read and approved the manuscript.

## Supplementary Material

Additional file 1: Table S1 List of *Trichoderma reesei* genes, which expression has been significantly changed in response to mycoparasitism to *R. solani.***Table S2.** List of *Trichoderma virens* genes, which expression has been significantly changed in response to mycoparasitism to *R. solani.***Table S3.** List of *Trichoderma atroviride* genes, which expression has been significantly changed in response to mycoparasitism to *R. solani.***Table S4.** Real Time PCR quantification of selected genes. Click here for file
